# Cytotoxicity studies of Fe_3_O_4_ nanoparticles in chicken macrophage cells

**DOI:** 10.1098/rsos.191561

**Published:** 2020-04-08

**Authors:** Shan Zhang, Shu Wu, Yiru Shen, Yunqi Xiao, Lizeng Gao, Shourong Shi

**Affiliations:** 1 Poultry Institute, Chinese Academy of Agricultural Sciences, Yangzhou, Jiangsu 225125, People's Republic of China; 2 Institute of Biophysics, Chinese Academy of Science, CAS Engineering Laboratory for Nanozyme, Institute of Biophysics, CAS, Beijing 100101, China; 3 Jiangsu Co-innovation Centre for Prevention and Control of Important Animal Infectious Diseases and Zoonoses, Yangzhou, Jiangsu 225000, People's Republic of China

**Keywords:** magnetic Fe_3_O_4_ nanoparticles, cytotoxic effects, oxidative index, apoptosis, chicken macrophage cells

## Abstract

Magnetic Fe_3_O_4_ nanoparticles (Fe_3_O_4_-NPs) have been widely investigated for their biomedical applications. The main purpose of this study was to evaluate the cytotoxic effects of different sizes of Fe_3_O_4_-NPs in chicken macrophage cells (HD11). Experimental groups based on three sizes of Fe_3_O_4_-NPs (60, 120 and 250 nm) were created, and the Fe_3_O_4_-NPs were added to the cells at different doses according to the experimental group. The cell activity, oxidative index (malondialdehyde (MDA), superoxide dismutase (SOD) and reactive oxygen species (ROS)), apoptosis and pro-inflammatory cytokine secretion level were detected to analyse the cytotoxic effects of Fe_3_O_4_-NPs of different sizes in HD11 cells. The results revealed that the cell viability of the 60 nm Fe_3_O_4_-NPs group was lower than those of the 120 and 250 nm groups when the same concentration of Fe_3_O_4_-NPs was added. No significant difference in MDA was observed among the three Fe_3_O_4_-NP groups. The SOD level and ROS production of the 60 nm group were significantly greater than those of the 120 and 250 nm groups. Furthermore, the highest levels of apoptosis and pro-inflammatory cytokine secretion were caused by the 60 nm Fe_3_O_4_-NPs. In conclusion, the smaller Fe_3_O_4_-NPs produced stronger cytotoxicity in chicken macrophage cells, and the cytotoxic effects may be related to the oxidative stress and apoptosis induced by increased ROS production as well as the increased expression of pro-inflammatory cytokines.

## 1. Introduction

The biomedical applications of superparamagnetic iron oxide nanoparticles (SPIONs) in magnetic resonance imaging, targeted therapy and cell labelling have been extensively studied. Although many applications in the diagnosis and treatment of diseases have shown good potential, there are still some controversial results concerning the cytotoxic effects from the use of SPIONs [1,2]. Some studies have reported that SPIONs are biologically benign [3], whereas other researchers believe that SPIONs have potential toxicity including organ toxicity and genotoxicity [1].

Magnetic Fe_3_O_4_ nanoparticles (Fe_3_O_4_-NPs) are a new type of nanomaterial with large specific surface area, high biocompatibility and biodegradability, which fall under the SPIONs category and have great potential for development for use in biomedicine [4]. Fe_3_O_4_-NPs have unique physical and chemical properties, including the following: superparamagnetism, magnetocaloric effects and peroxidase-like activity. The superparamagnetism of Fe_3_O_4_-NPs can be targeted to regulate Fe_3_O_4_-NPs *in vivo* through an external magnetic field for use in the targeted transport of drugs [5,6]. The magnetocaloric effect of Fe_3_O_4_-NPs can be used to convert their electromagnetic energy into heat energy through a repeated magnetization process, which can be used for tumour hyperthermia [7]. The peroxidase activity of Fe_3_O_4_-NPs can catalyse the degradation of H_2_O_2_ in acidic or neutral pH environments [8]. In recent years, Fe_3_O_4_-NPs have also shown great value by inhibiting bacterial activity [9–12]. Shi *et al.* [11] reported that the Fe_3_O_4_-NPs may be a potential antibiotic alternative to control *Salmonella enteritidis* infection during clinical therapy and in poultry industry operations. Based on the potential function of Fe_3_O_4_-NPs in disease diagnosis and treatment, it is necessary to further study the cytotoxic effects of Fe_3_O_4_-NPs before they are applied in various fields.

Although some reports on the cytotoxic effects of Fe_3_O_4_-NPs have been reported, most of them focus on the research in mammals (rats, mice or humans) [13–16]. There are few reports on the application of Fe_3_O_4_-NPs in animal husbandry [17,18]. There are zoonotic infectious diseases caused by bacteria such as *Salmonella enteritidis*, which has led to large numbers of deaths in humans and caused economic losses in animal husbandry. Our previous research found that Fe_3_O_4_-NPs could effectively control *S. enteritidis* infection in chicken LMH cells [11]. As macrophages play an important role in natural immunity and acquired immunity during *S*. *enteritidis* infection, we choose the macrophages of poultry as the research object.

Chicken HD11 macrophages are a kind of immortalized cell line formed by transforming chicken bone marrow cells through the replication-deficient avian leukaemia virus MC29 strain, which has been widely used in the study of infection immunity and its mechanism in bacteria or viruses [19]. HD11 cells share many similarities with normal chicken macrophages and have the advantage of rapid subculture growth; hence, they can be used as an ideal model of chicken macrophages for *in vitro* studies [20–22]. Moreover, poultry belongs to Aves and has many different characteristics in structure and function from mammals. Is there any difference between the application of Fe_3_O_4_-NPs in mammals and poultry? No research has reported such results yet. Therefore, we studied the cytotoxic effects of Fe_3_O_4_-NPs of different sizes on chicken macrophages in terms of their oxidative effects, apoptosis and pro-inflammatory cytokine secretion, laying a foundation for the potential application of Fe_3_O_4_-NPs as potential antibiotic substitutes in the poultry industry.

## 2. Material and methods

### 2.1. Characterization of Fe_3_O_4_ nanoparticles

Fe_3_O_4_-NPs with diameters of approximately 60, 120 and 250 nm were prepared by a hydrothermal method with FeCl_3_ and NaAc·3H_2_O as raw materials. A 0.4 or 0.6 g sample of FeCl_3_ and 3.6 g of NaAc·3H_2_O were added into the mixed solvent of 10 ml of glycol and 30 ml of diethylene glycol and stirred until fully dissolved by ultrasound, and these solutions were used to prepare 60 or 120 nm Fe_3_O_4_-NPs. A 0.82 g sample of FeCl_3_ and 3.6 g of NaAc·3H_2_O were added into 40 ml of glycol and stirred to achieve full dissolution by ultrasound, which was used to prepare the 250 nm Fe_3_O_4_-NPs. After complete dissolution, the mixture was transferred into a 50 ml Teflon-sealed autoclave and heated at 200°C for 12 h. After being naturally cooled to room temperature, the reactants were washed three times with water and three times with ethanol and then dried at 60°C for 6 h under a vacuum. The morphology, particle size and size distribution of the Fe_3_O_4_-NPs were measured using scanning electron microscopy (SEM) (S-4800, Japan) and transmission electron microscopy (TEM) (Tecnai G2 F30 S-TWIN, America). The diameters of the Fe_3_O_4_-NPs in the dispersion were determined using the dynamic light scattering (DLS) technique (Nano ZS90, England). The zeta potential of the Fe_3_O_4_-NPs was also measured using a laser particle size and zeta potential analyser (Nano ZS90, England).

### 2.2. Cell cultures

The chicken macrophage HD11 cells were gifted from Professor Jiao of Yangzhou University. The HD11 cells were cultured in 1640 medium (HyClone, Utah, USA) containing 10% foetal bovine serum (FBS) (Gibco, CA, USA) and 1% penicillin and streptomycin (Solarbio, Beijing, China) at 37°C and 5% CO_2_.

### 2.3. Cell viability assay

Cell viability was measured by the CCK-8 assay kit (Dojindo, Japan) according to the manufacturer's instructions. Briefly, HD11 cells were seeded at approximately 2×10^5^ cells ml^−1^ in 96-well plates and treated with 60, 120 and 250 nm Fe_3_O_4_-NPs for 24 h at concentrations of 50, 100, 200 and 400 µg ml^−1^. A 10 µl aliquot of CCK-8 reagent was added to each well, and the cells were incubated for another 2 h. The absorbance at 450 nm was measured with a microplate reader (Infinite M200 Pro, Tecan, Switzerland) after incubation.

### 2.4. Determination of the oxidative index malondialdehyde, superoxide dismutase and reactive oxygen species

HD11 cells in 24-well plates were incubated with different sizes and different concentrations of Fe_3_O_4_-NPs in 1640 medium with 10% FBS. After 24 h of exposure, the cell culture supernatant was collected and centrifuged at 1000 r.p.m. for 10 min in preparation to measure the malondialdehyde (MDA) and superoxide dismutase (SOD). The levels of MDA and SOD were measured by ELISA methods (Nanjing Jiancheng Bioengineering Institute, Nanjing, China). The absorbance of MDA/SOD was measured at 532 nm/450 nm with a microplate reader (Infinite M200 Pro, Tecan, Switzerland).

Reactive oxygen species (ROS) levels were determined by a reactive oxygen species assay kit (Beyotime Biotechnology Ltd., Shanghai, China). HD11 cells in 96-well plates were incubated with Fe_3_O_4_-NPs of different sizes and at different concentrations in 1640 medium with 10% FBS. After 24 h of exposure to the Fe_3_O_4_-NPs, the cells were incubated with 10 µM DCFH-DA in medium without FBS at 37°C and 5% CO_2_ for 30 min. Then, DCFH-DA was removed, and the cells were washed twice with PBS. The DCF fluorescence was monitored at 485 nm excitation and 520 nm emission with a microplate reader (Infinite M200 Pro, Tecan, Switzerland).

### 2.5. Determination of the extent of apoptosis

Apoptosis of the HD11 cells exposed to Fe_3_O_4_-NPs of various sizes and concentrations was measured using an Annexin V-FITC apoptosis analysis kit (BD, NJ, USA) according to the manufacturer's instructions. HD11 cells in 12-well plates were incubated with Fe_3_O_4_-NPs of different sizes at different concentrations in 1640 medium with 10% FBS. After 24 h of exposure, the cells were collected and washed twice with cold PBS while being stirred at 1000 r.p.m. for 5 min. Then, the cells were adjusted to a concentration of 1 × 10^6^ cells ml^−1^ with 1 × binding buffer. A total of 100 µl of cell suspension was placed in a 5 ml Falcon tube, and 5 µl of FITC Annexin V was added to each tube in the dark for 20 min at room temperature. Then, 5 µl of PI was added to each tube in the dark for 5 min, and 400 µl of 1 × binding buffer was added to each tube. All samples were analysed using flow cytometry (BD LSRFortessa™, USA) within 1 h.

### 2.6. Determination of interleukin-1*β* expression

HD11 cells in 12-well plates were incubated with Fe_3_O_4_-NPs of different sizes in 1640 medium with 10% FBS. After 24 h, the cells were collected using Trizol (Invitrogen, Carlsbad, CA) to extract the total RNA according to the manufacturer's protocol. RT-PCR was performed to quantitate interleukin-1*β* (IL-1*β*) mRNA using StepOnePlus^TM^ (Applied Biosystems by Life Technologies, USA). The primers of IL-1*β* were designed and synthesized by the Invitrogen Biotechnology Co., Ltd (Shanghai, China; table 1).

**Table 1.** Primer sequences for the target genes.

gene	primer position	primer sequence (5′→3′)	product size (bp)
B-actin	forward	ACACCCACACCCCTGTGATGAA	136
	reverse	TGCTGCTGACACCTTCACCATTC	
IL-1*β*	forward	TGGGCATCAAGGGCTACA	244
	reverse	TCGGGTTGGTTGGTGATG	

### 2.7. Statistical analysis

All results are presented as the mean ± standard error (s.e.). One-way analysis of variance (ANOVA) (multiple comparisons using LSD test) was used to evaluate the multiple comparisons among different sizes of Fe_3_O_4_-NPs groups. *p* < 0.05 indicated a significant difference.

## 3. Results and discussion

### 3.1. Synthesis and characterization of Fe_3_O_4_ nanoparticles

**Figure 1.**
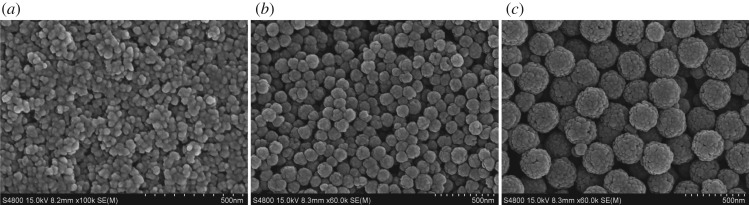
SEM images of Fe_3_O_4_-NPs. (*a*) 60 nm; (*b*) 120 nm; (*c*) 250 nm.

**Figure 2.**
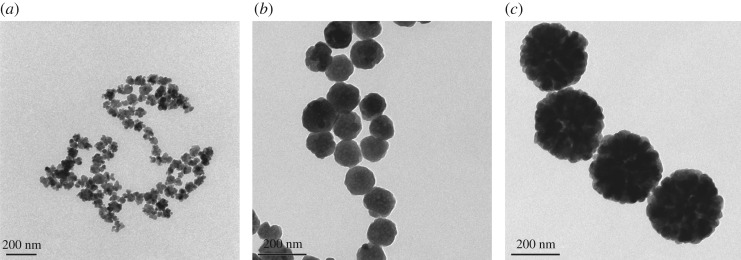
TEM images of Fe_3_O_4_-NPs. (*a*) 60 nm; (*b*) 120 nm; (*c*) 250 nm.

**Figure 3.**
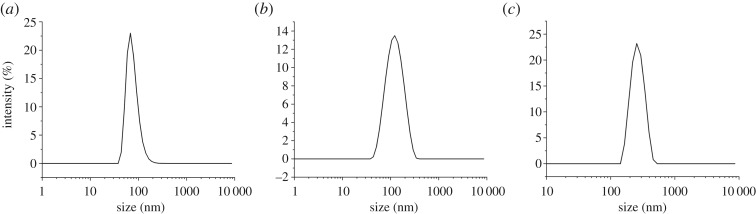
Zeta potential of Fe_3_O_4_-NPs. (*a*) 60 nm; (*b*) 120 nm; (*c*) 250 nm.

Micrographs obtained by SEM (figure 1) and TEM (figure 2) showed that the nanoparticles were spherical and uniform in shape. The hydrodynamic diameters of the Fe_3_O_4_-NPs were measured by DLS. As shown in figure 3, the average hydrodynamic diameters of the 60, 120 and 250 nm Fe_3_O_4_-NPs were 68 ± 2.40, 121.3 ± 3.04 and 250 ± 6.79 nm, respectively, which was in good agreement with the TEM results; the zeta potentials were +19.4 ± 1.52, +18.9 ± 1.48 and +20.3 ± 1.6 mV, respectively, indicating that the nanoparticles were positively charged, which is beneficial for endocytosis.

### 3.2. Cytotoxic effects of Fe_3_O_4_ nanoparticles of different sizes

**Figure 4.**
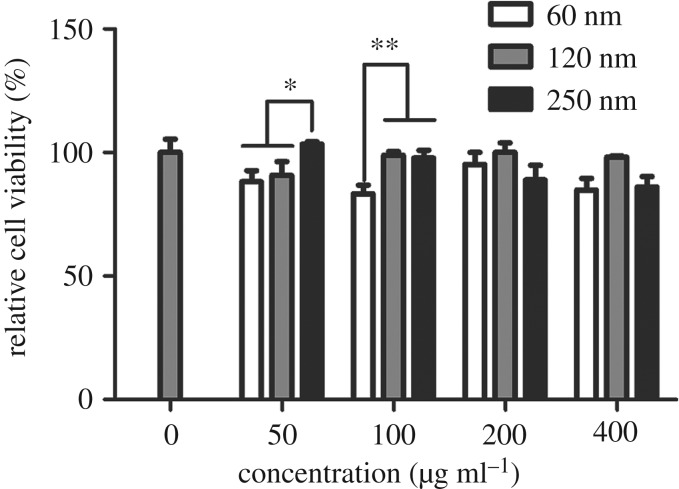
Effects of 60, 120 and 250 nm Fe_3_O_4_-NPs on the viability of HD11 cells. Control cells cultured in nanoparticle-free 1640 medium were processed in parallel to the treatment groups. The results of the CCK-8 assay were expressed as a percentage of the control. The data are expressed as the mean ± s.e., **p < 0.01, *p < 0.05. Fe_3_O_4_-NPs, magnetic Fe_3_O_4_ nanoparticles.

A CCK-8 assay was performed to evaluate the effect of different sized Fe_3_O_4_-NPs on HD11 cell viability. The HD11 cells were incubated with various concentrations of 60, 120 and 250 nm Fe_3_O_4_-NPs. As shown in figure 4, the cell viability of the 250 nm Fe_3_O_4_-NPs group was significantly greater than those of the 60 nm and 120 nm groups at the concentration of 50 µg ml^−1^, and the activity levels of the 120 nm and 250 nm groups were significantly greater than that of the 60 nm group at a concentration of 100 µg ml^−1^. These data suggested that the 60 nm Fe_3_O_4_-NPs produced a stronger cytotoxic effect when added at the same concentration as the other groups.

Many reports indicate that iron oxide nanoparticles are biologically safe because of their biocompatibility and high tolerance by cells [3,23,24]. However, other reports indicated that these nanoparticles had the potential to produce toxic effects in cells, and their toxic effects were related to their size, concentration, time, shape and the cell type [13,15,25,26]. The results from the present study suggest that the extent of Fe_3_O_4_-NPs toxic effects in HD11 cells is affected by the size of the Fe_3_O_4_-NPs. In our study, the 60 nm Fe_3_O_4_-NPs produced stronger cytotoxicity at the same concentration as the other groups according to the CCK-8 assay. By contrast, the 250 nm Fe_3_O_4_-NPs showed few cytotoxic effects in HD11 cells. These data suggested that the cytotoxic effects of small-sized Fe_3_O_4_-NPs were greater than those of large-sized Fe_3_O_4_-NPs for HD11 cells. Consistent with previous reports, Chen *et al.* [16] reported that smaller SPIONs produced stronger cytotoxicity in mouse bone marrow-derived macrophages. However, Gong *et al.* [25] evaluated 30 and 50 nm GoldMag nanoparticles (GMNPs) with SPIO as the core and gold coating, and they found that the nanotoxicity produced by the 50 nm GMNPs was significantly higher than that produced by 30 nm GMNPs on human umbilical vein endothelial cells when administered at identical levels of concentration and exposure. It may be that the size of iron nanoparticles compared in each experiment was different, and the different cells may have different responses to iron nanoparticles. In addition, the toxic effect of iron nanoparticles in cells is also related to the material with which they are coated. Therefore, it is important to fully investigate the biosafety of nanoparticles before they are used in biomedical or livestock production.

### 3.3. Effects of Fe_3_O_4_ nanoparticles on malondialdehyde, superoxide dismutase and reactive oxygen species levels

**Figure 5.**
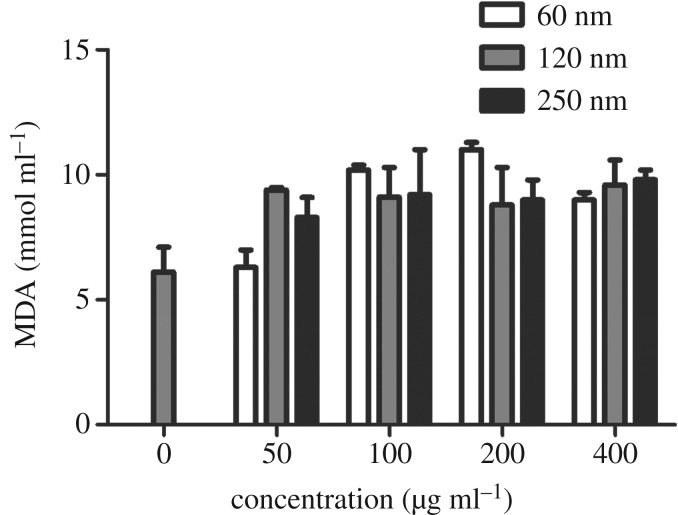
Effects of 60, 120 and 250 nm Fe_3_O_4_-NPs on MDA production in HD11 cells. Control cells cultured in nanoparticle-free 1640 medium were processed in parallel to the treatment groups. The data are expressed as the mean ± s.e. Fe_3_O_4_-NPs, magnetic Fe_3_O_4_ nanoparticles.

**Figure 6.**
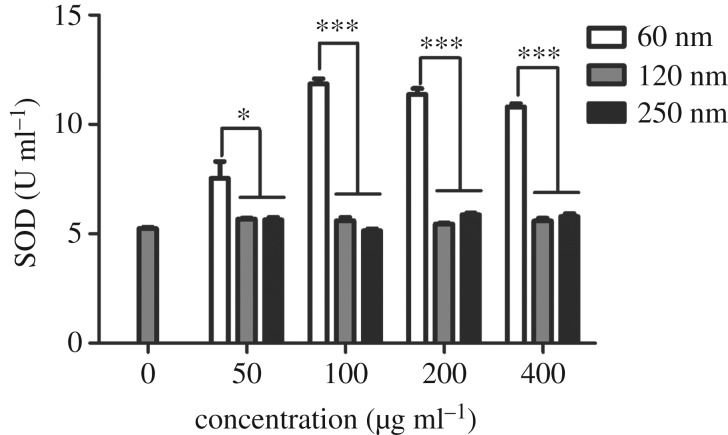
Effects of 60, 120 and 250 nm Fe_3_O_4_-NPs on SOD activities in HD11 cells. Control cells cultured in nanoparticle-free 1640 medium were processed in parallel to the treatment groups. The data are expressed as the mean ± s.e., ***p < 0.001, *p < 0.05. Fe_3_O_4_-NPs, magnetic Fe_3_O_4_ nanoparticles.

**Figure 7.**
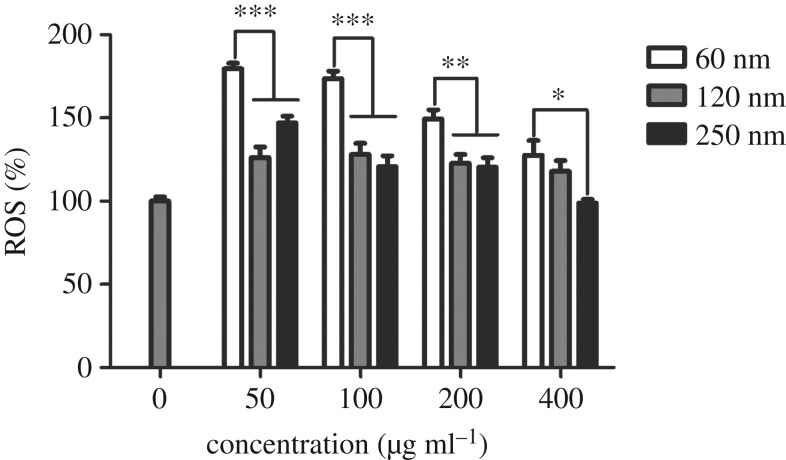
Effects of 60, 120 and 250 nm Fe_3_O_4_-NPs on ROS generation in HD11 cells. Control cells cultured in nanoparticle-free 1640 medium were processed in parallel to the treatment groups. The data are expressed as the mean ± s.e., ***p < 0.001, **p < 0.01, *p < 0.05. Fe_3_O_4_-NPs, magnetic Fe_3_O_4_ nanoparticles.

As shown in figure 5, no significant difference in the MDA level was observed among the three sizes of Fe_3_O_4_-NPs. However, the activity level of SOD differed, as presented in figure 6. HD11 cells showed significant increases in SOD activity when exposed to 60 nm Fe_3_O_4_-NPs. SOD levels of the 60 nm group were significantly higher than those of the 120 and 250 nm groups at 50 µg ml^−1^. SOD levels of the 60 nm group were significantly higher, showing extreme differences, than those of the 120 and 250 nm groups at 100, 200 and 400 µg ml^−1^. The results indicated that the 60 nm group had a significant effect on SOD levels, possibly due to the self-regulation of cells. Figure 7 showed the effects of different sizes of Fe_3_O_4_-NPs on ROS generation in HD11 cells. At concentrations of 50, 100 and 200 µg ml^−1^, ROS levels of the 120 and 250 nm groups were significantly lower, showing extreme differences, than those of the 60 nm group. The ROS level of the 250 nm group was significantly lower than that of the 60 nm group at 400 µg ml^−1^. These data suggested that the 60 nm Fe_3_O_4_-NPs produced more ROS when added at the same concentration as the other groups.

It has been reported that iron oxide nanoparticles induce cytotoxicity by activating the oxidative stress response [27,28]. The imbalance between oxidants and antioxidants favours the oxidants and potentially leads to damage known as ‘oxidative stress' [29]. MDA is the final decomposition product of membrane lipid peroxidation and widely used as a marker of oxidative lipid injury. SOD is a natural scavenger of oxygen-free radicals in organisms, which can eliminate ROS and inhibit the harmful effects of oxidant molecules on tissues and cells. ROS produced in biological systems play an important role in cell damage, cell apoptosis and other metabolic activities. Sarkar & Sil [28] found that iron oxide nanoparticles increased MDA and ROS levels in murine hepatocytes and decreased the activity of the antioxidant enzyme SOD, which correspondingly caused oxidative stress. In our study, we focused on the effects of different sizes of Fe_3_O_4_-NPs on cell oxidation. Compared with the control (nanoparticle-free) group, the three sizes of Fe_3_O_4_-NPs, 60, 120 and 250 nm, showed a trend of increasing MDA, but the MDA levels of the three sizes were not significantly different in our study. Among the three sizes, the SOD level of the 60 nm Fe_3_O_4_-NPs was the highest, whereas it was not enough to eliminate the production of ROS associated with Fe_3_O_4_-NPs of the same size because the ROS level of the group with Fe_3_O_4_-NPs of 60 nm was also the highest among the ROS levels measured in this study. The SOD levels for the Fe_3_O_4_-NPs of 120 and 250 nm were relatively low, possibly due to the compensatory reaction of the antioxidant system cells used to eliminate ROS production.

### 3.4. Effect of Fe_3_O_4_ nanoparticles on the extent of apoptosis

**Figure 8.**
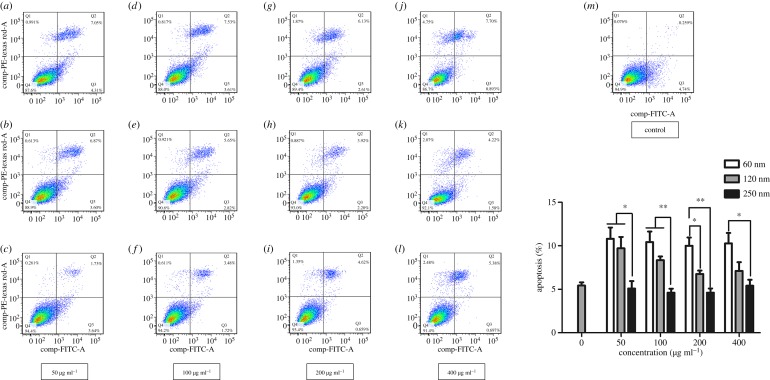
Effects of 60, 120 and 250 nm Fe_3_O_4_-NPs on the extent of apoptosis in HD11 cells. (*a*–*c*) 50 μg ml^−1^ of 60, 120 and 250 nm Fe_3_O_4_-NPs; (*d*–*f*) 100 μg ml^−1^ of 60, 120 and 250 nm Fe_3_O_4_-NPs; (*g*–*i*) 200 μg ml^−1^ of 60, 120 and 250 nm Fe_3_O_4_-NPs; (*j*–*l*) 400 μg ml^−1^ of 60, 120 and 250 nm Fe_3_O_4_-NPs; (*m*) the control group without nanoparticle. The data are expressed as the mean ± s.e., **p < 0.01, *p < 0.05. Fe_3_O_4_-NPs, magnetic Fe_3_O_4_ nanoparticles.

Figure 8 shows the effects of different sizes of Fe_3_O_4_-NPs on the extent of apoptosis of HD11 cells. At concentrations of 50 and 100 µg ml^−1^, the extent of apoptosis of the 250 nm group was significantly less than those of the 60 and 120 nm groups. At a concentration of 200 µg ml^−1^, the extent of apoptosis of the 120 nm group was significantly less than that of the 60 nm group, and the extent of apoptosis of the 250 nm group was extremely significantly less than that of the 60 nm group. Additionally, the extent of apoptosis of the 250 nm group was significantly lower than that of the 60 nm group at 400 µg ml^−1^. These data suggested that the extent of apoptosis was in the order 60 nm > 120 nm > 250 nm.

We observed that the greatest extent of apoptosis was caused by the 60 nm Fe_3_O_4_-NPs in this study. This finding was consistent with previous reports suggesting that the high levels of ROS could cause apoptosis or cell death [30,31]. When the production of intracellular ROS exceeds the threshold of cell ability for antioxidant defences, macromolecules such as DNA, proteins and lipids are damaged, which could lead to this pathophysiological state [32]. Therefore, the results of our study showed that the apoptosis induced by Fe_3_O_4_-NPs was mainly related to oxidative stress due to increased ROS production, and the apoptosis induced by small-sized Fe_3_O_4_-NPs was relatively more serious. However, there may be other pathways involved in apoptosis such that further study is needed.

### 3.5. Effect of Fe_3_O_4_ nanoparticles on IL-1*β* expression

**Figure 9.**
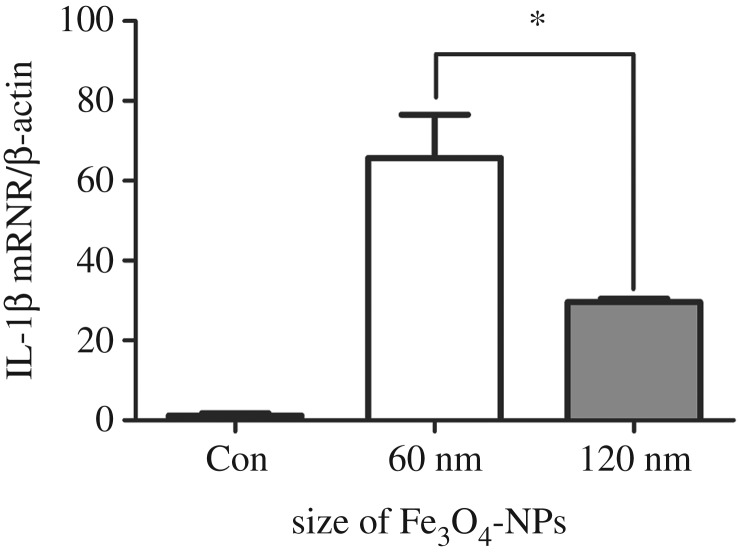
Effects of 60 and 120 nm Fe_3_O_4_-NPs at the concentration of 100 μg ml^−1^ on IL-1*β* expression of HD11 cells. Control cells cultured in nanoparticle-free 1640 medium were processed in parallel to the treatment groups. The data are expressed as the mean ± s.e., *p < 0.05. Fe_3_O_4_-NPs, magnetic Fe_3_O_4_ nanoparticles.

In addition, we suspect that the cytotoxicity of Fe_3_O_4_-NPs may be related to the secretion of inflammatory cytokines in macrophages. Based on the above results, the cell activity of the 60 nm group was significantly lower than those of the 120 and 250 nm groups at the concentration of 100 µg ml^−1^, and the biological safety of 250 nm Fe_3_O_4_-NPs was better. Therefore, we chose 60 and 120 nm Fe_3_O_4_-NPs at 100 µg ml^−1^ to detect their effects on inflammatory cytokines in macrophages. As shown in figure 9, the IL-1*β* secreted by macrophages was increased by 60 and 120 nm Fe_3_O_4_-NPs, and the IL-1*β* expression of the 60 nm group was the highest, which was consistent with the maximum cytotoxicity.

Macrophages are one of the main cells involved in the inflammatory response. IL-1*β* is one of the important inflammatory cytokines, which mediates the important process of inflammation [33]. Previous studies have reported that iron oxide nanoparticles could induce the cellular inflammatory response and increase the secretion of pro-inflammatory cytokines in human cells or mouse cell lines [34–36]. However, the overproduction of inflammatory cytokines could cause many inflammatory diseases, such as lung injury, obesity and diabetes [37,38]. It has also been reported that iron oxide nanoparticles could inhibit tumour growth by pro-inflammatory immune responses [39]. In general, the secretion of inflammatory cytokines by macrophages is a double-edged sword. On the one hand, it can cause cell damage, degeneration or death; on the other hand, it can enhance cell resistance to disease and make the intracellular and extracellular environment reach a new balance. In this study, we found that Fe_3_O_4_-NPs could increase the expression of IL-1*β* in HD11 cells, and the IL-1*β* expression was the highest when induced by 60 nm Fe_3_O_4_-NPs, which was consistent with the maximum cytotoxicity induced by 60 nm Fe_3_O_4_-NPs. This suggested that the inflammatory response may also be one of the important mechanisms for the cytotoxicity induced by Fe_3_O_4_-NPs.

## 4. Conclusion

In conclusion, the smaller Fe_3_O_4_-NPs produced stronger cytotoxicity in chicken macrophage cells, which may be related to the oxidative stress and apoptosis induced by the increased ROS production as well as the increased expression of pro-inflammatory cytokines. Based on this study, 250 nm Fe_3_O_4_-NPs have good biological safety, which lays a foundation for the potential application of Fe_3_O_4_-NPs in the poultry industry. The study of Fe_3_O_4_-NPs as potential antibiotic substitutes and their mechanism requires further experiments.

## Supplementary Material

Data supporting my paper

Reviewer comments

## Data accessibility

All the data are provided in the electronic supplementary material.
