# Correction to ‘Cytotoxicity studies of Fe_3_O_4_ nanoparticles in chicken macrophage cells’

**DOI:** 10.1098/rsos.200641

**Published:** 2020-05-13

**Authors:** Shan Zhang, Shu Wu, Yiru Shen, Yunqi Xiao, Lizeng Gao, Shourong Shi

*R. Soc. Open Sci.* **7**, 191561. (Published Online 8 April 2020). (doi:10.1098/rsos.191561)

This correction refers to an error in the funding statement. Grant number 31972587 for the National Natural Science Foundation of China had not been listed. This has now been corrected.

